# Development of a mathematical model for microbial consumption of glucose and fructose during cocoa (*Theobroma cacao* L.) fermentation process

**DOI:** 10.1128/spectrum.02387-24

**Published:** 2025-04-21

**Authors:** Daniel López-Puentes, Franyer López-Valiente, Daniel Rojas-Rodriguez, Diana Arias-Moreno

**Affiliations:** 1 Faculty of Basic Sciences, School of Biological Sciences, BIOPLASMA Research Group, Universidad Pedagógica y Tecnológica de Colombia 585528 , Tunja, Boyacá, Colombia; 2 Faculty of Basic Sciences, School of Mathematics, Universidad Pedagógica y Tecnológica de Colombia 28023 , Tunja, Boyacá, Colombia; 3 Faculty of Agricultural Engineering, Universidad del Tolima 28005, Ibague, Tolima, Colombia; University of Mississippi, University, Mississippi, USA

**Keywords:** model, fermentation, microorganisms, cocoa, simulation

## Abstract

A fundamental procedure for achieving the highest quality of cocoa is the fermentation of the bean carried out by microorganisms, a process in which the flavor and aroma of chocolate are developed. The objective of this research was to develop a mathematical model of microbial sugar consumption during the cocoa fermentation process. In this way, it is possible to obtain a mathematical tool that allows the representation of sugar consumption during the fermentation process. Initially, the need to construct a mathematical model was proposed due to its economic importance for small-scale producers in the national and international industries. In addition to the lack of control over the fermentation process, the complexity in terms of the microbial communities involved yeast (Y), lactic acid bacteria (LAB), and acetic acid bacteria (AAB). The methodological approach included (i) bibliographic review; (ii) specification of the parameters of the model, state, initial values, and equations; (iii) implementation of the mathematical model with the parameters, state, and initial values defined for each investigation; and (iv) validation of the model by comparing it with the experimental data from each publication. As a result, the model presented here for microbial sugar consumption during cocoa fermentation has the ability to represent each of the selected data points with significantly high precision. Furthermore, the proposed model aims to enhance the quality, efficiency, and sustainability of cocoa production, as well as contribute to scientific research related to this process. However, it is necessarily a simplification of the various biological processes that occur during cocoa bean fermentation.

**IMPORTANCE:**

Creating a mathematical model based on ordinary differential equations for glucose and fructose consumption by Y and LAB during cocoa fermentation is vital for advancing scientific and industrial practices. Cocoa fermentation is a complex, multi-step process involving microbial communities that play a key role in developing the flavor and aroma of chocolate. A robust mathematical model helps to understand the interactions between these microorganisms and sugar dynamics, providing a clearer understanding of the fermentation process.

## INTRODUCTION

Global cocoa production is concentrated in tropical nations in Africa, such as Côte d'Ivoire, Ghana, and Nigeria, and in Latin America, with notable producers including Ecuador, Colombia, and Brazil (1). According to the World Cocoa Foundation, between 40 and 50 million people worldwide depend on cocoa for their livelihoods (2). Currently, global production records 4.9 million tons of cocoa beans (3), with the selling price per ton ranging between $3,221 and $3,628 (4). Cocoa price volatility puts small producers in a vulnerable position (5). Undoubtedly, cocoa constitutes a significant source of income for small-scale producers who are responsible for the majority of global production (6). Furthermore, as a commercial crop, it can provide essential income for food acquisition (7), and it is particularly important in areas where food security has been problematic (8). Cocoa grown in Colombia is renowned for its high quality as fine aroma cocoa, a distinction that is significant, given that only 5% of cocoa produced and marketed globally is classified in this category. The country has the potential to increase production while maintaining this unique quality by enhancing production processes. Additionally, there is potential for establishing a value chain that could involve rural communities in both transformation and marketing processes (9).

Specifically, fermentation is considered the most critical step in the post-harvest processing of cocoa, as the beans are the main raw material in the chocolate industry (10). When the beans are removed from the fruit, the pulp is spontaneously inoculated with a variety of environmental microorganisms. In particular, the pulp provides an ideal medium for microbial growth due to its water and sugar content (11). At the beginning of the process, yeast (Y) carries out the fermentation of sugars present in the cocoa pulp, converting it into ethanol while performing pectinolysis and producing various flavor compounds such as higher alcohols, aldehydes, organic acids, and esters. On the other hand, lactic acid bacteria (LAB) ferment glucose (Glu), fructose (Fru), and citric acid from the cocoa pulp, transforming them into lactic acid, acetic acid, mannitol, and pyruvate. This creates a microbiologically stable fermentation environment and provides lactate as an essential carbon source for the growth of acetic acid bacteria (AAB) (12). Although the process is of high industrial relevance, there have been few attempts to construct a mathematical model of cocoa fermentation. Some of the reasons include the lack of control over the fermentation process itself, as well as the systemic complexity in terms of the microbial communities involved: Y, LAB, and AAB (13).

Mathematical models prove beneficial in predicting phenomena and supporting decision-making in various fields of study. Moreover, when applied and interpreted correctly, these models are highly useful (14), thereby avoiding potential significant costs in both industry and research. The cocoa fermentation process is inherently uncontrolled and varies significantly, leading to inconsistencies in flavor and quality (15). Different modeling approaches have been developed for the cocoa fermentation and drying process. These include both empirical and mechanistic models, which aim to describe the dynamics of fermentation based on various factors such as temperature, time, and microbial activity (13, 16–19). Kinetic modeling specifically addresses the rates of biochemical reactions during cocoa fermentation. This type of modeling allows for detailed insights into how different variables influence the production or degradation of different compounds, such as sugars, acids, or alcohols, which are vital for aroma and flavor development (15). Mathematical models of cocoa fermentation can be applied to design better fermentation processes, control operations, and predict production outcomes. By utilizing dynamic simulation tools, the cocoa industry can achieve greater efficiency and consistency in product quality (13, 15, 20). Consequently, the aim of this research was to develop a mathematical model of microbial sugar consumption during the cocoa fermentation process. In particular, to approach the issue, the research aimed to answer the question: what mathematical model represents the microbial consumption of glucose and fructose during the cocoa fermentation process?

## METHODS

### Bibliographic review

A literature review focused on studies of cocoa bean fermentation was conducted in order to gather experimental data (21–27). The assays analyzed in this research were documents published between 2007 and 2019. As inclusion criteria, only works written in English that recorded time series for the metabolites [Glu] and [Fru], in addition to having time series on the [Y] and [LAB] biomass community, were considered.

Information was collected from each selected study for the model, including the country, year of publication, cocoa variety, and fermentation method. The experimental data required for model implementation were compiled (Table 1). Different cocoa varieties and fermentation methods were considered, along with the initial fermentation conditions specific to each study (see Supplemental Material 2 at https://doi.org/10.5281/zenodo.14897702).

The values reported for the parameters used in the estimation of the model with glucose and fructose as substrates are presented in Tables S1 and S2 of the Supplemental Material (see Appendix 1 and Appendix 2 at https://doi.org/10.5281/zenodo.14897702), where the corresponding references are cited: (Moreno-Zambrano et al. (20), Bideaux et al. (28), Alfenore et al. (29), Sonnleitner & Käppeli (30), Kaspar von Meyenburg (31), Snoep et al. (32), Wang et al. (33), Aguilar-Uscanga et al. (34), Lee et al. (35), Berry et al. (36), Yoo et al. (37), Babel et al. (38), Verduyn et al. (39), Moustafa & Collins (40), Toran-Diaz et al. (41), Arroyo-López et al. (42), de Andrade Silva et al. (43), Yalçin & Özbaş (44), Yalçin & Özbaş (45), Vrancken et al. (46) Gustaw et al. (47).

**TABLE 1:** Experimental data selected for model simulation^*a*^^,*b*^

Data	Reference	Variety	Method
Brazil–WB1	(23)	Criollo/Forastero	Wooden box
Brazil–WB2	(23)	Criollo/Forastero	Wooden box
Ghana–H1	(21)	Criollo/Forastero	Heap
Dominican Republic–WB1	(22)	Trinitario	Wooden box
Ecuador–P1	(24)	Nacional/Trinitario	Platform
Ecuador–P2	(24)	Nacional/Trinitario	Platform
Ecuador–WB1	(24)	Nacional/Trinitario	Wooden box
Ecuador–WB2	(24)	Nacional/Trinitario	Wooden box
Brazil–PB1	(27)	NA	Plastic box
Brazil–T1	(27)	NA	Tank
Malaysia–WB1	(25)	Mixed hybrids	Wooden box
Nicaragua–WB1	(26)	Nugu/O’payo	Wooden box
Nicaragua–WB2	(26)	Nugu/O’payo	Wooden box

^*a*^ Each entry in the table represents a distinct fermentation experiment characterized by the following parameters. Country: Identifies the nation where the fermentation experiment was conducted. Code: A unique identifier assigned to each study, reflecting the fermentation method employed. For instance, "H" denotes heap fermentation, "WB" signifies wooden box fermentation, "P" indicates platform fermentation, "PB" refers to plastic box fermentation, and "T" represents tank fermentation. Variety: Specifies the cacao variety utilized in the fermentation process. Notably, "N/A" is used when such information is not available. Fermentation Method: Describes the specific technique applied during the fermentation, such as wooden box, heap, platform, plastic box, or tank methods. Reference: Cites the original study from which the experimental data were sourced, providing proper attribution and enabling readers to consult the primary research for more detailed information.

^*b*^ Code assigned to each study based on the fermentation method: e.g., Ghana–H1 and Brazil–WB1 (21, 23), respectively.

### Model specifications

A mathematical model was conceptualized where the degradation of sugars [Glu] and [Fru] by the community of microorganisms [Y] and [LAB] formed the basis of the cocoa fermentation model (see Supplemental Material 1 at https://doi.org/10.5281/zenodo.14897702). According to Moreno-Zambrano et al. (13), the physical-chemical conditions, such as temperature, pH, humidity, dissolved soluble solids, oxygen concentration, and titratable acidity, were not taken into account.

Ordinary differential equations (ODEs) (48) were used to describe the consumption of glucose and fructose by microorganisms during the cocoa fermentation process. To do this, ODE was formulated for representing the microbial kinetics (yield coefficient $Y_{\frac{microorganism}{sustrate}}$ and growth rate $\nu_{c}$), the presence of the community microorganisms involved, [Y] and [LAB], and the consumption of sugars [Glu] or [Fru] by these microorganisms (49).

During the characteristic microbial growth, four phases are easily recognized (lag phase, exponential phase, stationary phase, and death phase). However, the cocoa fermentation process has shown a partial lack of the lag phase and an almost complete absence of a stationary phase for all microbial populations involved. Hence, cocoa fermentation, microbial growth of the [Y] and [LAB] communities can be expressed as a combination of the change in the microbial community over time between the exponential growth phase and the death phase (20). Thus,


(1.1) $$\frac{d\left[Y\right]}{dt}=\;\nu_{c}-v_{d}$$

(1.2) $$\frac{d\left[LAB\right]}{dt}=\;\nu_{c}-v_{d}$$

where [Y] and [LAB] represent the biomass of the microbial community, and $\nu_{c}\;and\;v_{d}$ are equations describing the growth and mortality rates of the microbial community, respectively. Specifically, the growth rate equation $\nu_{c}$ for the exponential phase can be formulated (13) as follows:


(1.3) $$\nu_{c}=\mu \left[Y\right]$$

(1.4) $$\nu_{c}=\mu \left[LAB\right]$$

where μ represents the specific growth rate of a particular microorganism. An important aspect for modeling the process is to limit microbial growth through nutrient abundance [Glu] or [Fru]. One of the models that considers this is the Contois equation (49), which assumes a nutrient limiting the growth rate:


(1.5) $$\mu =\frac{\mu_{max}\left[S\right]}{K_{s}\left[Y\right]+\left[S\right]}$$

(1.6) $$\mu =\frac{\mu_{max}\left[S\right]}{K_{s}\left[LAB\right]+\left[S\right]}$$

In this case, μ represents the specific microbial growth rate; $\mu_{max}$ is the maximum specific growth rate; [*S*] is the substrate or rate-limiting nutrient [Glu] or [Fru]; and $K_{s}$ is the substrate saturation constant. This interpretation from (15) allows expression of equations 1.3 and 1.4 as:


(1.7) $$\nu_{c}=\frac{\mu_{max}\left[S\right]}{K_{s}\left[Y\right]+\left[S\right]}\left[Y\right]$$

(1.8) $$\nu_{c}=\frac{\mu_{max}\left[S\right]}{K_{s}\left[LAB\right]+\left[S\right]}\left[LAB\right]$$

In this case, the Contois equation is considered under the assumption that the growth rate of the [Y] and [LAB] communities also depends on their size. Another assumption that can be made about the Contois equation is that the consumed nutrients are immediately used to increase microbial biomass. In this sense, in terms of microbial community growth, the yield coefficient $Y_{\frac{Y}{Glu}}$ can be defined as the ratio between the change in yeast biomass (∆Y) and the change in substrate concentration (∆Glu) or glucose as follows:


(2.1) $$Y_{\frac{Y}{Glu}}=-\;\frac{\frac{d\left[Y\right]}{dt}}{\frac{d\left[Glu\right]}{dt}}$$

Solving for $\frac{d\left[Glu\right]}{dt}$and replacing $\frac{d\left[Y\right]}{dt}$ with equation 1.1,


(3.1) $$\frac{d\left[Glu\right]}{dt}=-\;\frac{1}{Y_{\frac{Y}{Glu}}}\frac{d\left[Y\right]}{dt}=\;-\;\frac{1}{Y_{\frac{Y}{Glu}}}\left(\nu_{c}-\nu_{d}\right)$$

According to Moreno-Zambrano (20), since dead cells cannot utilize any substrate to produce products, then $\nu_{d}$ in equation 3.1 reduces to 0. Consequently, the ODE describing the amount of glucose consumed by Y can be formulated as follows:


(3.2) $$\frac{d\left[Glu\right]}{dt}=-\;\frac{1}{Y_{\frac{Y}{Glu}}}\left(\nu_{c}-0\right)=-\;Y_{\frac{Glu}{Y}}\nu_{c}=-\beta_{1}$$

In this specific case, if one also considers that [LAB] participate in glucose consumption during fermentation, equation 3.2 is rewritten as follows:


(3.3) $$\frac{d\left[Glu\right]}{dt}=-\;Y_{\frac{Glu}{Y}}\nu_{c}-\;Y_{\frac{Glu}{LAB}}\nu_{c}=-\beta_{1}-\beta_{2}$$

where ${-\beta }_{1}$ and $-\beta_{2}$ are constants associated with glucose consumption by [Y] and [LAB] communities, respectively. This is based on the premise that each consumption constant –β at time *t* is directly proportional to the substrate amount [Glu] at time *t*; that is, the consumption rate –β for each involved microbial community is proportional to the substrate amount at time *t* and based on the principle that physical quantities should be consistent in terms of units. Then, the dependent variable [Glu] and a dimensional fermentation constant *λ* (mg·g^−1^) are added. Finally, equation 3.3 is rewritten as follows:


(4.1) $$\frac{d\left[Glu\right]}{dt}=-\frac{\beta_{1}}{\lambda }\left[Glu\right]-\frac{\beta_{2}}{\lambda }\left[Glu\right]$$

In the case of the ODE for fructose, thus:


(4.2) $$\frac{d\left[Fru\right]}{dt}=-\frac{\beta_{3}}{\lambda }\left[Fru\right]-\frac{\beta_{4}}{\lambda }\left[Fru\right]$$

Below is the analytical solution for each of the formulated ODEs, equation 4.1 and 4.2 respectively:


$$\frac{d\left[Glu\right]}{dt}=-\frac{Y_{\frac{Glu}{Y}}\nu_{1}}{\lambda }\left[Glu\right]-\frac{Y_{\frac{Glu}{LAB}}\nu_{2}}{\lambda }\left[Glu\right]$$

$$\frac{d\left[Glu\right]}{\left[Glu\right]}=-\frac{Y_{\frac{Glu}{Y}}\nu_{1}}{\lambda }dt-\frac{Y_{\frac{Glu}{LAB}}\nu_{2}}{\lambda }dt$$

$$\int \frac{1}{\left[Glu\right]}d\left[Glu\right]=-\int \frac{Y_{\frac{Glu}{Y}}\nu_{1}}{\lambda }dt-\int \frac{Y_{\frac{Glu}{LAB}}\nu_{2}}{\lambda }dt$$

$$\int \frac{1}{\left[Glu\right]}d\left[Glu\right]=-\frac{Y_{\frac{Glu}{Y}}\nu_{1}}{\lambda }\int dt-\frac{Y_{\frac{Glu}{LAB}}\nu_{2}}{\lambda }\int dt$$

$$Ln\left(\left[Glu\right]\right)=-\left(\frac{Y_{\frac{Glu}{Y}}\nu_{1}}{\lambda }t+\frac{Y_{\frac{Glu}{LAB}}\nu_{2}}{\lambda }t\right)+c$$

$$Ln\left(\left[Glu\right]\right)=-\left(\frac{Y_{\frac{Glu}{Y}}\nu_{1}}{\lambda }+\frac{Y_{\frac{Glu}{LAB}}\nu_{2}}{\lambda }\right)t+c$$

$$\left[Glu\right]\left(t\right)=e^{-\left(\frac{Y_{\frac{Glu}{Y}}\nu_{1}}{\lambda }+\frac{Y_{\frac{Glu}{LAB}}\nu_{2}}{\lambda }\right)t+c}$$

$$\left[Glu\right]\left(t\right)=e^{-\left(\alpha \right)t+c}$$

Considering that the concentration of [Glu] at *t = 0* is equal to the maximum concentration of glucose in the cocoa pulp or the glucose at the beginning of fermentation, it is possible to approach the equation as an initial value problem or Cauchy problem, as follows:


$$t=0,\;\;then:\;\left[Glu\right]\left(0\right)={\left[Glu\right]}_{max}={\left[Glu\right]}_{initial}$$

Therefore, solving for c,


$$\left[Glu\right]\left(0\right)=e^{-\left(\alpha \right)t+c}=e^{-\left(\alpha \right)t}e^{c}$$

$$\left[Glu\right]\left(0\right)=e^{-\left(\alpha \right)0}e^{c}=e^{c}$$

$$\left[Glu\right]\left(0\right)={\left[Glu\right]}_{max}=e^{c}$$

$$Ln\left({\left[Glu\right]}_{max}\right)=c$$

Replacing c


$$\left[Glu\right]\left(t\right)=e^{-\left(\frac{Y_{\frac{Glu}{Y}}\nu_{1}}{\lambda }+\frac{Y_{\frac{Glu}{LAB}}\nu_{2}}{\lambda }\right)t+Ln\left({\left[Glu\right]}_{max}\right)}$$

$$\left[Glu\right]\left(t\right)=e^{-\left(\frac{Y_{\frac{Glu}{Y}}\nu_{1}}{\lambda }+\frac{Y_{\frac{Glu}{LAB}}\nu_{2}}{\lambda }\right)t}{\left[Glu\right]}_{max}$$

Finally, the particular analytical solution of the glucose ODE:


(4.3) $$\left[Glu\right]\left(t\right)=e^{-\left(\alpha \right)t}{\left[Glu\right]}_{max}$$

In the same way (see Supplemental Material 1 at https://doi.org/10.5281/zenodo.14897702), the particular analytical solution of ODE for fructose is obtained:


(4.4) $$\left[Fru\right]\left(t\right)=e^{-\left(\alpha \right)t}{\left[Fru\right]}_{max}$$

### Model implementation

Then, to specify the model, the necessary parameters were estimated to implement the model of sugar consumption in each of the selected studies, taking into account the definition of the differential equation. The estimated parameters were the yield coefficient, $Y_{\frac{microorganism}{substrate}}$, the maximum growth rate (*µ*_max_) of each microorganism (50), and the substrate concentration at which *µ*_max_ is half or *K*_*s*_ (20). The parameter estimation is represented for each metabolite as follows: glucose (see Appendix 1 at https://doi.org/10.5281/zenodo.14897702) and fructose (see Appendix 2 at https://doi.org/10.5281/zenodo.14897702). Both estimations take into account the initial [Y] and [LAB] biomass communities (see Supplemental Material 3 at https://doi.org/10.5281/zenodo.14897702). Likewise, the average, standard deviation, and values reported in the literature for each estimated parameter are presented. The estimation of the model parameters was sensitive to the quantity and accuracy of the data reported in each of the collected studies. Since all selected articles report the size of the [Y] and [LAB] microbial communities in colony-forming units, the conversion factor chosen for the [Y] community was 15 pg·CFU^−1^ equivalent to *Saccharomyces cerevisiae* (51) and 1.25 pg·CFU^−1^ for the [LAB] community equivalent to *Lactobacillus plantarum* (20). The concentration of [Glu] and [Fru] as the biomass of the microbial community (Y and LAB) in the model is presented in units (mg·g^−1^ [pulp]). The initial values of each study were considered for the modeling process (initial substrate, mg·g^−1^, and initial biomass, mg.g^−1^), and the boundary conditions (time, *t*) were limited using the last data point of the reported time series in each research.

These ODEs, along with the model constraints, initial conditions, and boundary conditions, were adapted for implementation in RStudio software version 2023.03.1 + 446 using the deSolve package version 1.35 (52) and the ggplot2 package version 3.4.1 (53). Each ODE was solved 10,000 times per 24 hour interval (see Supplemental Material 2 at https://doi.org/10.5281/zenodo.14897702).

### Model validation

The validation of the model was carried out using experimental data from the literature, including different cocoa varieties (Criollo, Forastero, Trinitario and Others) and various fermentation techniques: boxes, heap, tank, and platforms (54–57). To verify the accuracy of the model, a regression analysis was applied to compare the model results with the experimental data. This was done using the coefficient of determination (*R*^2^) (58). Additionally, the root mean square error (RMSE) was determined to quantify the magnitude of deviation of simulated values from observed values (59). A higher *R*^2^ value indicates a better fit of the model to the data, while a lower RMSE indicates that the model better represents the observed data (58, 59).

The results obtained from the implementation were analyzed, and the model’s ability to represent the experimental data were evaluated. Comparative statistical methods were used to establish differences between predictions and experimental data, including the Kolmogorov-Smirnov (K-S) two-sample test with a *D* test statistic and a 95% confidence interval (60). A comparison was made between the results obtained from the model and the experimental data from the literature using a hypothesis test with a significance level of α = 0.05 (61). Additionally, the numerical solution of one ODE was fitted to different regression models: (i) linear, (ii) polynomial, (iii) logistic, and (iv) non-linear (Fig. 2), using the ggtrendline package version 1.0.3 (62) to verify the analytical solution $\left[Glu\right]\left(t\right)=e^{-\left(\alpha \right)t}{\left[Glu\right]}_{max}$. Finally, in Fig. 2d, a non-linear regression model $y\;\left(x\right)={ae}^{-bx}$ was adjusted to the numerical solution of the ODE glucose provided in RStudio software. Here, *a* and *b* are estimated parameters from the data provided and match with the parameters of the proposed analytical solution ${\left[Glu\right]}_{max}$ and -α, respectively.

## RESULTS

### Model outcomes

**Fig 1:**
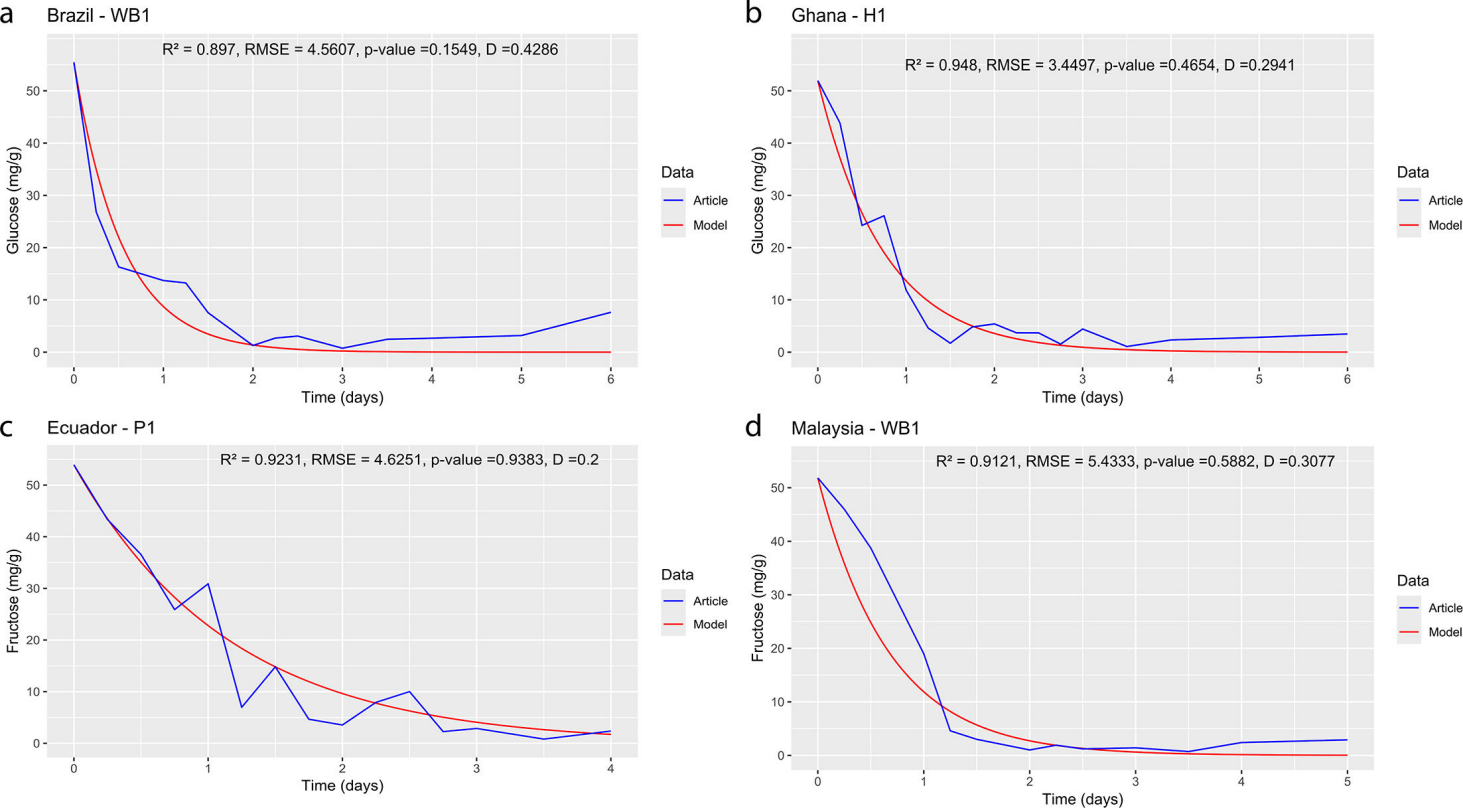
Graphical representation of the model against the experimental data. (**a**) Glucose (23), (**b**) Glucose (21), (**c**) Fructose (24), and (**d**) Fructose (25). Panels (a) and (b) show model simulations for glucose consumption, with experimental data from (23) and (21), respectively. Panels (c) and (d) display the corresponding simulations for fructose consumption using datasets from (24) and (25). The high coefficients of determination (*R*² values of 0.897, 0.948, 0.9231, and 0.9121) along with low RMSE values demonstrate that the model accurately captures the dynamics of sugar depletion. Moreover, the Kolmogorov-Smirnov tests yielded test statistics of D = 0.4286, 0.2941, 0.2, and 0.3077 with *P*-values exceeding 0.05, supporting the null hypothesis that there is no significant difference between the experimental data and the model predictions. These results confirm that the underlying assumptions and parameter estimations—based on experimental conditions and microbial growth kinetics—are robust and align well with observed fermentation behavior.

The results of the model implementation demonstrated that different cocoa varieties and fermentation methods significantly influenced the fermentation outcomes (Fig. 1). Specifically, the fermentation methods and cocoa varieties used in the Brazil–WB1 (23) and Nicaragua–WB1 (26) studies (Table 1) resulted in the highest yield coefficient $Y_{\frac{microorganism}{substrate}}$ for [Glu], with values of 225.32 mg·mg^−1^ for [Y] and 1,145.05 mg·mg^−1^ for [LAB], respectively. In addition, the highest yield coefficient $Y_{\frac{microorganism}{substrate}}$ for [Fru] was observed in the Brazil–WB2 (23) and Nicaragua–WB1 (26) studies, with values of 239.00 mg·mg^−1^ for [Y] and 1,450.80 mg·mg^−1^ for [LAB], respectively (see Appendix 1 and Appendix 2 at https://doi.org/10.5281/zenodo.14897702). The high values of the yield coefficient $Y_{\frac{microorganism}{substrate}}$ in the cocoa fermentation process, as observed in the selected studies (23, 26), indicate a remarkable efficiency of the microorganisms in converting substrates (63), [Glu] and [Fru], into microbial biomass, [Y] or [LAB]. Likewise, the average yield coefficients $Y_{\frac{microorganism}{substrate}}$ for fructose, *X̅* = 87.92 mg·mg^−1^ (*σ* = 76.63 mg·mg^−1^) and *X̅* = 272.86 mg·mg^−1^ (*σ* = 409.83 mg·mg^−1^), are even higher than for glucose *X̅* = 75.32 mg·mg^−1^ (*σ* = 60.75 mg·mg^−1^) and *X̅* = 230.31 mg·mg^−1^ (*σ* = 314.11 mg·mg^−1^), suggesting that microorganisms may be better adapted or may prefer fructose as an energy source in the conditions studied (see Appendix 1 and Appendix 2 at https://doi.org/10.5281/zenodo.14897702). Also in this case, the yield coefficients for the [LAB] community show the highest values both for the glucose [Glu] and [Fru] substrates, indicating that the [LAB] community might be better suited than the [Y] community for the consumption of sugars (see Supplemental Material 3 at https://doi.org/10.5281/zenodo.14897702). This indicates that the [LAB] community is much more efficient than the others communities for the consumption of [Glu] and [Fru] in these studies (64). Additionally, model results (Fig. 1) demonstrated that certain initial fermentation conditions (see Supplemental Material 2 at https://doi.org/10.5281/zenodo.14897702) like initial substrate [Glu] and [Fru], or initial microbial biomass [Y] and [LAB], influenced rate sugar consumption during the cocoa bean fermentation process (see Supplemental Material 3 at https://doi.org/10.5281/zenodo.14897702).

Regarding *µ*_max_, the highest values of *µ*_max_ for glucose and fructose (0.7575 h^−1^ for Y and 0.6343 h^−1^ for LAB) were observed in the same study, Ecuador–P2 (24). A comparison of the *µ*_max_ values for Y and LAB reveals distinct dynamics in their roles during the fermentation process (see Appendix 1 and Appendix 2 at https://doi.org/10.5281/zenodo.14897702). Specifically, the average *µ*_max_ for glucose was *X̅* = 0.3144 h⁻¹ (*σ* = 0.2135 h⁻¹) for yeast and *X̅* = 0.3498 h⁻¹ (*σ* = 0.1603 h⁻¹) for LAB, while for fructose, the values were *X̅* = 0.3205 h⁻¹ (*σ* = 0.2052 h⁻¹) for yeast and *X̅* = 0.3498 h⁻¹ (*σ* = 0.1603 h⁻¹) for LAB. These results indicate that most of the values of *µ*_max_ are within the optimal ranges in the studied conditions (Table 1) for both Y and LABs (20). Only the *µ*_max_ values from the studies Brazil–WB1 (23), Ecuador P2 (24), and Nicaragua WB1 (26) were outside the reported range for glucose (see Appendix 1 and Appendix 2 at https://doi.org/10.5281/zenodo.14897702). The out-of-range *µ*_max_ values were 0.01592, 0.7575, and 0.01247 h^−1^ for Brazil–WB1 (23), Ecuador P2 (24), and Nicaragua WB1 (26), respectively. All other reported *µ*_max_ values (see Appendix 1 and Appendix 2 at https://doi.org/10.5281/zenodo.14897702) fall within the reported range (20).

With respect to the *K*_*s*_ values, all estimated values for the glucose data set (see Appendix 1 at https://doi.org/10.5281/zenodo.14897702) are slightly outside the range reported in the literature for each case (20). However, all estimated *K*_*s*_ values for the fructose data set (see Appendix 2 at https://doi.org/10.5281/zenodo.14897702) align with those reported in the literature (20). Specifically, the average *K*_*s*_ for glucose was *X̅* = 0.6160 mg·mg^−1^ (*σ* = 0.05108 mg·mg^−1^) for yeast and *X̅* = 0.6102 mg·mg^−1^ (*σ* = 0.06112 mg·mg^−1^) for LAB, while for fructose, the values were *X̅* = 0.6268 mg·mg^−1^ (*σ* = 0.03429 mg·mg^−1^) for yeast and *X̅* = 0.6422 mg·mg^−1^ (*σ* = 0.04115 mg·mg^−1^) for LAB.

### Model validation

As evidenced by the results (Fig. 1), the current model for microbial sugar consumption during cocoa bean fermentation (see Supplemental Material 1 at https://doi.org/10.5281/zenodo.14897702) demonstrates the capability to accurately represent each data set of glucose (Fig. 1a and b) and fructose (Fig. 1c and d) with significantly high precision (see Supplemental Material 3 at https://doi.org/10.5281/zenodo.14897702). This implies that the assumptions formulated in this research are in line with the available biological knowledge to a considerable degree, as demonstrated in all simulations performed (see Supplemental Material 2 at https://doi.org/10.5281/zenodo.14897702). The model presented here is based on ODEs with initial conditions (Cauchy problem) and is capable of representing the concentration of sugars during cocoa fermentation and fitting to the available experimental data to an acceptable degree. However, it is necessarily a simplification of the various biological processes that occur during cocoa bean fermentation. Specifically, the values *R*^2^ = 0.897, *R*^2^ = 0.948, *R*^2^ = 0.9231, and *R*^2^ = 0.9121 reported in the studies (21, 23–25) demonstrate one of the best fits achieved by the model in the different glucose and fructose simulations (Fig. 1). Additionally, the RMSE values of 4.5607 (23), 3.4497 (21), 4.6251 (24), and 5.4333 (25) are presented to compare the error in the same units of the dependent variable (mg·g^−1^). Finally, the test statistic *D* = 0.4286, *D* = 0.2941, *D* = 0.2, and *D* = 0.3077 (Fig. 1) and *P* > 0.05 of the K-S test in each of the studies (21, 23–25) allow acceptance of the null hypothesis (Ho): there is no significant difference between the data reported by the article and the data provided by the model (see Supplemental Material 2 at https://doi.org/10.5281/zenodo.14897702).

**Fig 2:**
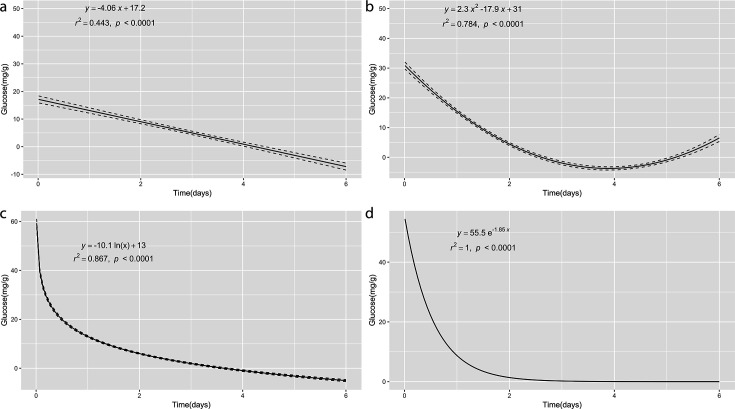
Regression Analysis of the ODE Numerical Solution. Adjustment of the one numerical solution of the ODE to different regression models. (a) Linear regression model: y (x)=−ax+b. (b) Polynomial regression model: $\;y\;\left(x\right)={ax}^{2}-bx+c$. (c) Logistic regression model: y (x)=−aln(x)+b. (d) Non-linear regression model: $y\;\left(x\right)={ae}^{-bx}$ that presents the best fit and matches with the proposed analytical solution $\left[Glu\right]\left(t\right)=e^{-\left(\alpha \right)t}{\left[Glu\right]}_{max}$ . Data: Glucose, Brazil–WB1 (23). Representation of the degree of fit between the numerical solution of the proposed ordinary differential equation (ODE) model and various regression models applied to the same dataset. Panel (a) shows the linear regression, (b) the polynomial regression, (c) the logistic regression, and (d) the non-linear regression model. Notably, the non-linear regression in panel (d) achieves an optimal fit (*R*² = 1), closely matching the proposed analytical solution. In this panel, the estimated parameter "a"—which represents the initial glucose concentration—is approximately 55.5 mg·g⁻¹ (SE = 3.68e-07), while parameter "b", reflecting the combined rate of glucose consumption by yeasts and lactic acid bacteria, is estimated at about -1.85 (SE = 1.72e-08). The near-zero p-value (<2e-16) and a very small residual standard error (1.33e-06) underscore the statistical significance and precision of these estimates, thereby reinforcing the model's capacity to accurately describe the sugar consumption dynamics during cocoa fermentation.

In particular, Fig. 2 shows the degree of adjustment of the numerical solution of the ODE proposed by RStudio software against different regression models (see Supplemental Material 2 at https://doi.org/10.5281/zenodo.14897702). The regression models chosen were (i) linear regression model, y (x)=−ax+b; (ii) polynomial regression model, $\;y\;\left(x\right)={ax}^{2}-bx+c$; (iii) logistic regression model, y (x)=−aln(x)+b; and (iv) non-linear regression model, $y\;\left(x\right)={ae}^{-bx}$, which present the best fit and match with the proposed analytical solution $\left[Glu\right]\left(t\right)=e^{-\left(\alpha \right)t}{\left[Glu\right]}_{max}$. The values of *R*^2^ demonstrate the degree of adjustment of the numerical solution in relation to different regression models: linear regression model, *R*^2^ = 0.443; polynomial regression model, *R*^2^ = 0.784; logistic regression model, *R*^2^ = 0.867; and non-linear regression model, *R*^2^ = 1. In Fig. 2d, the estimated value for parameter *a* in a non-linear regression model $y\;\left(x\right)={ae}^{-bx}$ is approximately 55.5 with a standard error of 3.68e-07. This value represents the [Glu] concentration (mg·g^−1^) at the beginning of fermentation in the study by Papalexandratou et al., 2011. The estimated value for the parameter *b* is approximately −1.85 with a standard error of 1.72e-08. This value represents the combined rate of glucose consumption -(α) by [Y] and [LAB]. These estimated values are the best-fitting parameters according to the non-linear model. The standard errors indicate the precision of these estimates. The *P* value is virtually 0 (<2e-16), suggesting that both parameters are highly significant in the model (see Supplemental Material 2 at https://doi.org/10.5281/zenodo.14897702). The residual standard error is a measure of how well the model fits with data; in this case, it is very small (1.33e-06), indicating a good fit of the model to the data.

In the case of the non-linear regression model (Fig. 2d), the coefficient of determination (*R*^2^) is 1; this value is explained by the fact that the proposed analytical solution, $\left[Glu\right]\left(t\right)=e^{-\left(\alpha \right)t}{\left[Glu\right]}_{max}$, is adjusted and matched with the non-linear regression model, $y\;\left(x\right)={ae}^{-bx}$, to calculate parameters *a* and *b* in the fermentation process. Parameters *a* and *b* coincide with the initial substrate concentration [Glu] and the rate of consumption of the substrate, -(α), respectively. This indicates a perfect fit of the non-linear model $y\;\left(x\right)={ae}^{-bx}$ to the numerical solution that allows the correct calculation of these parameters and corroborates the proposed analytical solution, $\left[Glu\right]\left(t\right)=e^{-\left(\alpha \right)t}{\left[Glu\right]}_{max}$. Moreover, the *F* statistic is used to assess the overall significance of the model. In this case, the value of the *F* statistic is extremely large (3.79e + 16), and the *P* value is 0, suggesting that the non-linear model overall is highly significant (see Supplemental Material 2 at https://doi.org/10.5281/zenodo.14897702). Ultimately, the sum of the squared residuals is a measure of the discrepancy between the observed values and the values predicted by the model. In this case, it is extremely small (1.06e-09), suggesting a good fit of the non-linear regression model, $y\;\left(x\right)={ae}^{-bx}$. In summary, this result indicates that the non-linear regression model $y\;\left(x\right)={ae}^{-bx}$ fits very well to the proposed analytical solution, $\left[Glu\right]\left(t\right)=e^{-\left(\alpha \right)t}\left[Glu\right]{}_{max}$, and the numerical solution provided by RStudio software (see Supplemental Material 2 at https://doi.org/10.5281/zenodo.14897702), with highly significant parameters and a high capacity to explain the variability in the data (Fig. 2d).

## DISCUSSION

Mathematical modeling in biology offers valuable insights but faces significant limitations. Models are constrained by current scientific knowledge and can only indicate possibilities, not historical realities (65). They often abstract critical elements from biological systems, potentially oversimplifying complex processes (65, 66). The disconnect between modeling and experimental biology hinders full integration, necessitating a cultural shift toward interdisciplinary collaboration (67). In sloppy systems, where many parameters are practically unidentifiable, optimal experimental design may inadvertently introduce systematic errors, making models less predictive (68). Despite these challenges, mathematical modeling remains crucial for understanding complex biological systems across scales, from molecular to ecological levels (66, 67). To overcome limitations, researchers should consider hierarchies of models with varying complexity and focus on identifying underlying mechanisms rather than precise parameter estimation (68). The presented model focuses on the dynamics of glucose and fructose consumption by yeasts and lactic acid bacteria but does not explicitly incorporate critical environmental factors such as humidity, temperature, or the total microbial community composition. These variables are known to influence fermentation outcomes, and their exclusion represents a limitation of the current work.

Mathematical models have been developed to understand and improve cocoa bean fermentation, a complex process crucial for chocolate quality. These models typically incorporate microbial growth, substrate utilization, and metabolite production (13, 16). Recent advancements include exploring additional biochemical mechanisms (15). Some models can also account for intrinsic and extrinsic fermentation parameters, including pH, temperature, and oxygen levels (69). While these models provide valuable insights into fermentation dynamics and can help differentiate between experimental conditions (15), they still have limitations. For instance, some models struggle to fully explain microbial population dynamics (16) and abiotic parameters (13). To maintain the model’s tractability and interpretability, we simplified the equations by excluding certain environmental and microbial variables. While this approach allowed us to focus on the fundamental interactions between microorganisms and sugar dynamics, we recognize that it may limit the model’s applicability to real-world fermentation scenarios. Nevertheless, mathematical modeling offers a promising approach for understanding and optimizing cocoa bean fermentation, potentially leading to improved chocolate quality and more standardized production processes.

Recent research on cocoa fermentation and processing has focused on developing models to optimize production and quality. López‐Pérez et al. (18) created a kinetic model for traditional cocoa fermentation using genetic algorithms, which could aid in scaling up the process. Mathematical models for drying fermented cocoa beans were explored by Olabinjo and Olajide (70), identifying some optimal models for drying. Tosto et al. (71) reviewed existing cocoa models, highlighting gaps in plant processes, 3D architecture, and management practices while emphasizing the need for more data and collaboration. In this sense, John et al. (72) employed an experimental approach to model cocoa bean fermentation, revealing some key factors influencing bean components. Their study also demonstrated significant factor interactions, particularly regarding substrate/product role. These advancements in cocoa modeling contribute to improving production processes and understanding cocoa bean responses to various conditions.

The results obtained in this study confirm and expand upon previous findings in the literature regarding cocoa fermentation modeling, particularly in terms of the efficiency of substrate conversion into microbial biomass (13, 20). As observed, the yield coefficients, $Y_{\frac{microorganism}{substrate}}$, reported for glucose and fructose in the Brazil–WB1, Brazil–WB2, and Nicaragua–WB1 fermentation studies are notably high, indicating a high efficiency in converting these substrates into biomass (63). This aligns with previous studies that emphasize the importance of microbial adaptation to different fermentation conditions (21–27). The higher yield observed in the conversion of fructose compared to glucose suggests that microbial communities, particularly LAB, may be better adapted to or prefer fructose as an energy source under the conditions studied. This is consistent with previous research that suggests resource-based competition between [Y] and [LAB] during cocoa fermentation (13).

The values of *μ*_max_ and *K*_*s*_ obtained show variability, depending on the geographical origin and specific fermentation conditions. Comparing these values with the literature reveals that while most fall within reported ranges, some out-of-range values may indicate significant differences in microbial dynamics according to the fermentation environment, which is consistent with previous studies (13, 20). The observed differences in *μ*_max_ and *K*_*s*_ values suggest that specific fermentation conditions (such as temperature, humidity, and microbiome composition) can significantly influence the results, limiting the generalizability of the model to unstudied conditions. However, the high accuracy of the proposed model, as evidenced by high *R*² values and low RMSE, indicates that the underlying biological assumptions are robust and that the model can accurately represent the collected experimental data. Additionally, the model’s fit to analytical solutions and its comparison with different regression models further reinforce the validity of the approach adopted in this study.

Although the developed model shows a good fit to the experimental data, it is important to recognize that it necessarily simplifies the biological processes involved. Modeling based on ODEs does not fully capture the complexity of microbial interactions and environmental variations that may occur during fermentation. However, mathematical models allow coherence to be given to the vast amount of experimental or field data obtained from the study of nature. These models provide guidance and insight into the crucial variables that need to be evaluated, as well as a method for interpreting that data (73). The model presented here evaluates and confirms the importance of the microbial community (Y and LAB) during the sugar fermentation process in cocoa pulp. In particular, cocoa bean fermentation is spontaneous and involves microorganisms from workers’ hands, fruit surfaces, tools used in post-harvest processes, insects, banana leaves, trays, or boxes used in a previous fermentation (74). However, not all of these microorganisms participate in the fermentation process (21). Specifically, the microbial succession involved in the cocoa bean fermentation process is mainly represented by Y, LAB, and AAB (10). In this regard, it would be valuable to develop models that explicitly include multiple microbial species and communities, considering their interactions and competition. Such models could provide a more detailed understanding of the microbial dynamics during fermentation.

Moreover, mathematical models can generate predictions of a specific biological phenomenon. Biology advances more efficiently by focusing on verifying or discarding specific predictions rather than indiscriminate measurements without a defined purpose (73). The model presented allows evaluation of the degree of adjustment using the *R*^2^ value and to verify if there is a significant difference between the model and the experimental data with the K-S test and *P* value (58–60). Although the values of the test statistic *D* may indicate a certain degree of difference between the mathematical model and the experimental data (e.g., *D* = 0.4286 and *D* = 0.3077 suggest a moderate difference), the fact that *P* values are greater than 0.05 means that these differences could be due to random variation and not an actual discrepancy between the model and the data. Similarly, it is possible to quantify the degree of error through the RMSE estimator. Thus, the model has the capability to represent the consumption of glucose and fructose by Y and LAB during cocoa fermentation, as well as quantify the degree of error and significant difference compared to the field-evaluated data. In particular, future research should focus on incorporating environmental factors such as temperature and humidity into mathematical models to better reflect the variability observed in cocoa fermentation processes. Although the model demonstrates high accuracy, further validation through controlled experiments under different fermentation conditions would be beneficial to confirm and refine the model parameters. In this regard, the model presented does not allow for the prediction of fermentation quality or correct biological succession of key microorganisms. It is also necessary to recognize that it is impossible to predict with this model the final quality of the fermentation process. To ensure a preliminary assessment of the model’s performance, we validated it using experimental data from the literature, encompassing diverse fermentation conditions, cocoa varieties, and techniques. While this approach provided a robust initial validation, we acknowledge that dedicated experiments under controlled conditions are necessary for a more comprehensive evaluation.

We explicitly acknowledge that the validation of the model using experimental data from the literature represents a limitation of the current study. While this approach provided a preliminary assessment of the model’s performance, it does not replace the need for controlled, parallel fermentation experiments to confirm its accuracy and applicability in real-world scenarios. The model’s structure is flexible and can be extended to include additional variables, such as temperature-dependent reaction rates, humidity effects, or shifts in microbial community composition. Future work will focus on incorporating these factors to enhance the model’s accuracy and predictive power.

### Conclusions

The model based on ODEs with initial conditions is capable of fitting the available experimental data to an acceptable degree. However, it is important to note that this model is a necessary simplification of the various biological processes involved in cocoa bean fermentation.

In conclusion, although the model system developed by ODEs provides a detailed mathematical approach to the fermentation process, its practical application has limitations. The simplicity of the predictions generated by the model contrasts with the complexity of the system. The inability of these models to accurately predict fermentation quality or appropriate biological succession of relevant organisms suggests that a more integrative and practical approach is needed. It is crucial to continue developing predictive tools that are not only theoretically robust but also effective and applicable in real scenarios, particularly to improve efficiency and results in small-scale and industrial fermentation processes.

Finally, the small discrepancies between the model predictions and the experimental data, as well as parameter values outside the ranges, indicate that aspects that also influence the fermentation process have not been taken into account.

## Data Availability

All supplemental materials associated with this study have been deposited in Zenodo and are available at https://doi.org/10.5281/zenodo.14897702. This repository includes the mathematical model (Supplemental Material 1), the scripts used in the analysis (Supplemental Material 2), the study figures (Supplemental Material 3), as well as Tables S1 and S2 (Appendices 1 and 2, respectively).
